# Parasitism of Lepidopterous Stem Borers in Cultivated and Natural Habitats

**DOI:** 10.1673/031.011.0115

**Published:** 2011-02-14

**Authors:** Duna Madu Mailafiya, Bruno Pierre Le Ru, Eunice Waitherero Kairu, Stéphane Dupas, Paul-André Calatayud

**Affiliations:** ^1^ Unité de Recherche IRD 072, International Centre of Insect Physiology and Ecology (ICIPE), PO Box 30772, Nairobi, Kenya or Université Paris-Sud 11,91405 Orsay cedex, France; ^2^ Department of Zoological Sciences, School of Pure and Applied Sciences, Kenyatta University, PO Box 43844, Nairobi, Kenya; ^3^ Unité de Recherche IRD 072, CNRS, Laboratoire Evolution, Génomes et Spéciation, Bât 13, BP I, Avenue de la Terrassse, 91 198 Gif-sur-Yvette cedex, France et Université Paris-Sud 11, 91405 Orsay cedex, France

**Keywords:** agroecological zones, cereals, habitat types, seasons, wild host plants

## Abstract

Plant infestation, stem borer density, parasitism, and parasitoid abundance were assessed during two years in two host plants, *Zea mays* (L.) (Cyperales: Poaceae) and *Sorghum bicolor* (L.) (Cyperales: Poaceae), in cultivated habitats. The four major host plants (*Cyperus* spp., *Panicum* spp., *Pennisetum* spp., and *Sorghum* spp.) found in natural habitats were also assessed, and both the cultivated and natural habitat species occurred in four agroecological zones in Kenya. Across habitats, plant infestation (23.2%), stem borer density (2.2 per plant), and larval parasitism (15.0%) were highest in maize in cultivated habitats. Pupal parasitism was not higher than 4.7% in both habitats, and did not vary with locality during each season or with host plant between each season. *Cotesia sesamiae* (Cameron) and *C. flavipes* Cameron (Hymenoptera: Braconidae) were the key parasitoids in cultivated habitats (both species accounted for 76.4% of parasitized stem borers in cereal crops), but not in natural habitats (the two *Cotesia* species accounted for 14.5% of parasitized stem borers in wild host plants). No single parasitoid species exerted high parasitism rates on stem borer populations in wild host plants. Low stem borer densities across seasons in natural habitats indicate that cereal stem borer pests do not necessarily survive the non-cropping season feeding actively in wild host plants. Although natural habitats provided refuges for some parasitoid species, stem borer parasitism was generally low in wild host plants. Overall, because parasitoids contribute little in reducing cereal stem borer pest populations in cultivated habitats, there is need to further enhance their effectiveness in the field to regulate these pests.

## Introduction

In sub-Saharan Africa stem borers are major biotic constraints to cereal production. These pests are responsible for losses ranging between 5–73% of potential yield under different agroecological conditions (Seshu Reddy and Walker 1990; De Groote 2002; De Groote et al. 2003). Upon hatching, with the exception of *Sesamia calamistis* that bore directly into the stem (Bosque-Pérez and Schulthess, 1998), the first instar larvae of most stem borer species initially feed on young leaf tissues, while older larvae tunnel into the stem tissues and feed internally (Nye 1960; Bosque-Pérez and Schulthess 1998). Depending on the species, the larval stage may last 25–58 days and may have 6–8 instars. Pupation normally takes 5–14 days after which adult moths emerge (Harris 1990; Holloway 1998; Maes 1998). In maize, stem borers pupate close to the tunnel exit or even partly outside the stem (Smith et al. 1993), which increases their accessibility to parasitoids (Ndemah et al. 2001). On the contrary, in wild host plants, stem borers seldom pupate within plant stems, but rather on the outside often at the bottom of plants close to the roots in the soil (Mailafiya 2009).

These stem borers are attacked by a diverse group of both indigenous and exotic parasitoids (Bonhof et al. 1997; Overholt 1998; Zhou et al. 2003; Mailafiya et al. 2009). It is assumed that parasitism is higher on stem borer populations residing in wild grass communities than on those in cultivated crops due to: (1) non-periodic re-colonization of natural habitats by parasitoids (Conlong 1994), and (2) slow stem borer larval growth rates which increases their temporal window of susceptibility to stage-specific parasitoids (Bowden 1976; Overholt 1998). Parasitization of cereal stem borer pests during the non-cropping (off) season may therefore occur mainly in natural habitats (Schulthess et al. 1997).

Cereal crops are usually grown in small fields surrounded by land occupied by wild host plants of lepidopterous stem borers. Natural habitats have high stem borer diversity (Le Ru et al. 2006a, b), and thus, serve as refugia for sustaining parasitoid diversity within the ecosystem (Ndemah et al. 2002; Mailafiya et al. 2009, 2010). Also, across different ecological regions and seasons, stem borer parasitism is generally positively correlated with parasitoid richness and abundance (Mailafiya et al. 2010). Herbivore parasitism, however, can vary with habitat type depending on the prevailing conditions in a given ecosystem (Landis et al. 2000; Altieri and Nicholls 2004). Hence, it is imperative to understand the ecological role (i.e., herbivore pest population regulation) of natural habitats as a component of the cereal cropping system.

In Kenya, more than 95 parasitoid (Bonhof et al. 1997; Zhou et al. 2003; Mailafiya et al. 2009), 88 stem borer (Khan et al. 1997a; Le Ru et al. 2006a, b; Mailafiya et al. 2009), and 66 host plant (Le Ru et al. 2006a, b; Mailafiya et al. 2009) species have been recorded. However, only laboratory studies have assessed stem borer parasitism in both cereals and wild host plants (Sétamou et al., 2005), and these have particularly focused on one parasitoid, *Cotesia flavipes*; one stem borer, *Chilo partellus*; two cultivated cereals, *Zea mays* and *Sorghum bicolor,* and two wild host plants, *Pennisetum trachyphyllum* and *Sorghum arundinaceum.*

This study assessed the field parasitism of lepidopterous stem borers in various host plant genera found in cultivated habitats (*Z*. *mays* L. and *S.* *bicolour* L. (Poales: Poaceae)) and natural habitats (*Cyperus* spp., *Panicum* spp., *Pennisetum* spp. and *Sorghum* spp.) during different seasons in four agroecological zones in Kenya. Results obtained can provide crucial information on stem borer parasitism during the off season, or hint at the importance of parasitoids in regulating stem borer populations in different habitats. Ultimately, these should advance basic understanding of the ecological role of natural habitats as reservoir(s) for parasitoids during the off season.

## Materials and Methods

### Survey sites description

Field surveys were conducted over two years (from December 2005 to December 2007) in four localities representing different agroecological zones in Kenya: Suam (Trans-Nzoia District) in the highland tropics, Kakamega (Kakamega District) in the moist transitional agroecological zones, Mtito Andei (Makueni District) in the dry mid-altitudes, and Muhaka (Kwale District) in the lowland tropics.

Suam (1° 11′ N, 34° 47′ E, 1995 MASL) has a single cropping season that lasts from March to November. Average annual rainfall and temperature are 1190 mm and 19°C, respectively (Africa AWhere-ACT Database 2002). Local vegetation is characterized by a mosaic of both rain forest and secondary grassland. Suam is a major production region, where 50% of the area is under cereal cultivation at commercial scale mainly with an average field size of 3.4 ha. The area under natural habitats was 50%, of which the total relative cover of all potential wild host plants of stem borers were 11.2% and 10.9% during the rainy and dry seasons, respectively (Otieno et al. 2008).

Kakamega (0° 13′ N, 34° 56′ E, 1655 MASL) and has a bimodal rainfall distribution with two main cropping seasons occurring from March to August and October to December. Average annual rainfall and temperature are 1570 mm and 21° C, respectively (Africa AWhere-ACT Database 2002). The vegetation mosaic is of the Guineo-Congolian rain forest type. Kakamega is a moderate production region, with 43.3% of the area under cereal cultivation. Cereals were grown at subsistence level with an average field size of 0.28 ha located in open forest patches, or scattered around non-compact homesteads and along forest edges and the river bank. The area of natural habitats was 51.9%, of which the total relative cover of all potential wild host plants of stem borers were 0.5% and 0.3% during the rainy and dry seasons, respectively (Otieno et al. 2006).

Mtito Andei (2° 39′ S, 38° 16′ E, 760 MASL) has a single cropping season lasting from November to January. Average annual rainfall and temperature are 665 mm and 23° C, respectively (Africa AWhere-ACT Database 2002). The vegetation consists of Somalia-Masai Acacia-Commiphora deciduous bushland and thicket. Mtito Andei is a minor production region with cereals grown at subsistence level. Area under cereal cultivation was 27.3%, with an average field size of 0.37 ha mainly found surrounding sparsely populated and distant homesteads. The area of natural habitats was 72.7%, of which the total relative cover of all potential wild host plants of stem borers were 13.0% and 8.0% during the rainy and dry seasons, respectively (Otieno et al. 2008).

Muhaka (4° 18′ S, 39° 31′ E, 40 MASL) has a bimodal rainfall distribution with two main cropping seasons typically occurring from April to August and from October to December. Average annual rainfall and temperature are 1210 mm and 26° C, respectively (Africa AWhere-ACT Database 2002). Local vegetation is the East African coastal grassy and woody mosaic bordering the undifferentiated Zanzibar-Inhambane forest type. Muhaka is a moderate growing region with about 10.7% of the area under cereal cultivation, and an average field size of 0.15 ha. Cereals were grown at subsistence level, in fields scattered around a more compact homestead settlement. The area of natural habitats was 72.3%, of which the total relative cover of all potential wild host plants of stem borers were 2.2% and 1.0% during the rainy and dry seasons, respectively (Otieno et al. 2006).

In Kakamega and Muhaka, cereal crops were planted during the dry season in marshy areas usually bordering streams or rivers. Also, in localities with a single cropping season irrigated crops were found in Mtito Andei, but not in Suam.

### Data collection

Previous studies revealed that stem borer densities were much lower in wild host plants than in adjacent cultivated cereals (Gounou and Schulthess 2004; Ndemah et al. 2007; Matama-Kauma et al. 2008). Therefore, to increase the chances of collecting stem borer parasitoids from different habitats a random sampling scheme was used in cultivated habitats, and both random and non-random sampling schemes were used in natural habitats.

**Random sampling in cultivated habitats.** Based on the sampling plan developed by Overholt et al. (1994) and the proportion of land under cultivation (Guihéneuf 2004; Goux 2005) 21, 16, 16, and 10 cereal fields were randomly sampled in Kakamega, Mtito Andei, Muhaka, and Suam, respectively. In order to capture parts of early- to mid-whorl (vegetative [4 – 6 weeks]) and late-whorl to tasseling (reproductive [8 – 10 weeks]) stages of plant growth, every field was visited at least twice during each rainy and dry season. To estimate plant infestation, stem borer densities, and parasitism rates (depending on the field size and crop availability during different seasons) 50 to 100 plants were randomly sampled per field (Overholt et al. 1994). The plants collected were dissected in the field, and stem borer larvae or pupae obtained were reared in the laboratory on artificial diet for subsequent recovery of parasitoids.

**Random and non-random sampling in natural habitats.** To evaluate plant infestation or stem borer densities and parasitism rates in natural habitats, random and non-random sampling schemes were applied, respectively.

### Random sampling scheme

Grass patches immediately surrounding each sampled cereal field were visited at regular intervals during both dry and rainy seasons as stated above for cultivated habitats. Based on the sampling plan developed by Gounou and Schulthess (2004) to estimate plant infestation and stem borer densities, 50 to 100 plants or tillers (depending on the availability of host plant species during different seasons or due to disturbances [i.e. livestock grazing]) were randomly sampled per plant species at each sampling point, up to 50 m distance from the edge of each cereal field. Each plant or tiller collected was dissected in the field. Stem borer larvae or pupae obtained were reared in the laboratory on artificial diet for subsequent recovery of parasitoids.

### Non-random sampling scheme

Stem borers living in wild host plants were collected using the non-random sampling procedure applied by Le Ru et al. (2006a, b). During each sampling occasion as described above wild host plants exhibiting infestation symptoms were sampled, where possible up to 100 m of each cereal field was sampled. Depending on both field and crop size, 50 to 100 plants were randomly checked after each 10–15 steps taken in a zigzag manner. At each sampling site, all known host plants belonging to the Poaceae, Cyperaceae, Typhaceae, and Juncaceae families (Le Ru et al. 2006a, b) were inspected for infestation symptoms such as scarified leaves, dead hearts, entrance/exit holes, and frass. Percent parasitism was determined by dividing the number of parasitized larvae/pupae by the total number of larvae/pupae collected (Zhou et al. 2003).

### Stem borer parasitoid recovery

Stem borer larvae recovered were reared on artificial diet developed by Onyango and Ochieng-Odero (1994) in glass vials (2.5 cm diameter × 7.5 cm depth) plugged with cotton wool, which were kept under ambient conditions (26 ± 3° C; 65 ± 5 RH) in the laboratory until puparia or cocoon formation. Parasitoid puparia or cocoons recovered from stem borer larvae or pupae were kept separately in plastic vials (2.5 cm diameter × 7.5 cm depth) until adult emergence. Adult stem borer or parasitoid specimens were preserved in 70 % alcohol.

### Data analyses

Least mean squares following logistic regression was used to analyze percentage plant infestation (the proportion of plants with stem borers), stem borer density (the number of stem borers per plant), and percentage parasitism (the proportion of parasitized stem borer larvae, pupae, and their total) amongst localities and host plant genera or between seasons per habitat type. All data were analyzed using the Generalized Linear Model (PROC GENMOD; SAS 2001), to cater for binomial error distribution (Collett 1991). Significance was set at *P* ≤ 0.05.

## Results

### Species occurrence and dominance

In this study 10,195 stem borers were collected, of which 7,439 (from six species) and 2,756 (from 13 species) individuals were from cultivated and natural habitats, respectively. Also, 18 and 19 parasitoid species were recovered from stem borer hosts living in cultivated cereals and wild host plants, respectively. The details of parasitoid species found and their multitrophic interactions have been provided in Mailafiya et al. (2009). Stem borers and parasitoids were recovered from two (*Z*. *mays* and *S.* *bicolor)* and 16 (*Cymbopogon* spp., *Cynodon* spp., *Cyperus* spp., *Digitaria* spp., *Echinochloa* spp., *Eleusine* spp., *Eriochloa* spp., *Euclaena* spp., *Panicum* spp., *Pennisetum* spp., *Rottbellia* spp., *Schoenoplectus* spp., *Scleria* spp., *Setaria* spp., *Sorghum* spp., and *Typha* spp.) host plant genera in cultivated and natural habitats, respectively. However, due to insufficient collections (data replications) for most host plant genera, analysis in this study was limited to the following: the sedge, *Cyperus* spp. (Poales: Cyperaceae), and the grasses: *Panicum* spp., *Pennisetum* spp., and *Sorghum* spp. (Poales: Poaceae), as they not only occurred in most or all localities (like the cultivated cereals), but also had adequate replications.

Depending on the locality and host plant, *Busseola fusca, Chilo partellus, Sesamia calamistis* were the dominant stem borer species in cultivated cereals, while *Busseola phaia, Busseola* nov sp. 1, *Chilo orichalcociliellus, Ematheudes* sp., *Sesamia nonagrioides* were the dominant stem borer species in natural host plants (Table 1). Altogether, percentage stem borer species dominance were computed for 3 and 13 host plant species in cultivated and natural habitats, respectively. Unfortunately, due to very scanty (< 5 individuals) recovery (of single stem borer species each), percentage stem borer species dominance were not computed for *Eleusine corocana,* Sorghum arundinaceum, *Schoenoplectus maritimus* in Kakamega; *Echinochloa colonum, Eleusine corocana, Eleusine jaegeri, Eriochloa meyerana* in Mtito Andei; *Digitaria* sp., *Pennisetum* spp. in Muhaka; and *Cymbopogon nardus,* Cynodon sp., *Schoenoplectus confusus,* Setaria *incrassata* in Suam.

**Table 1.**
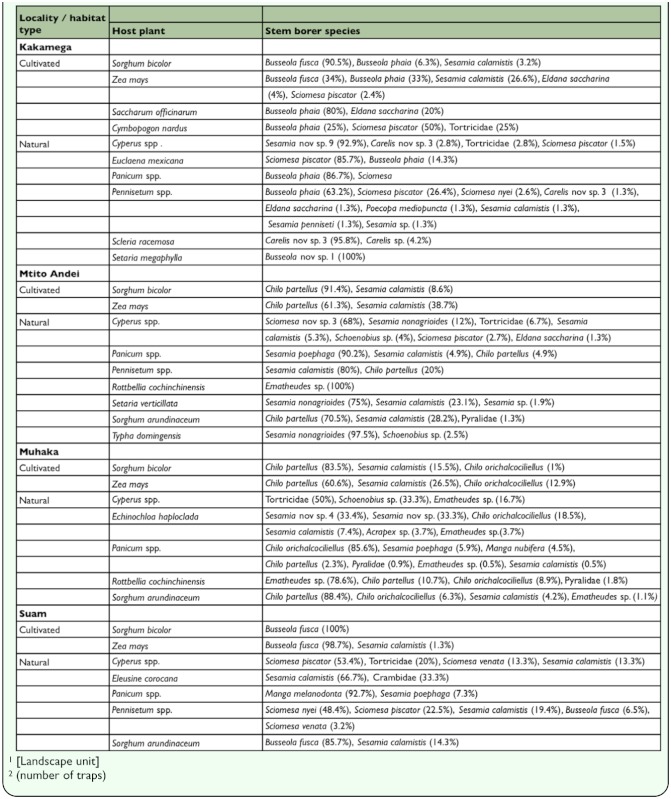
Stern borer dominance in various host plants in cultivated and natural habitats from four localities in Kenya

**Table 2.**
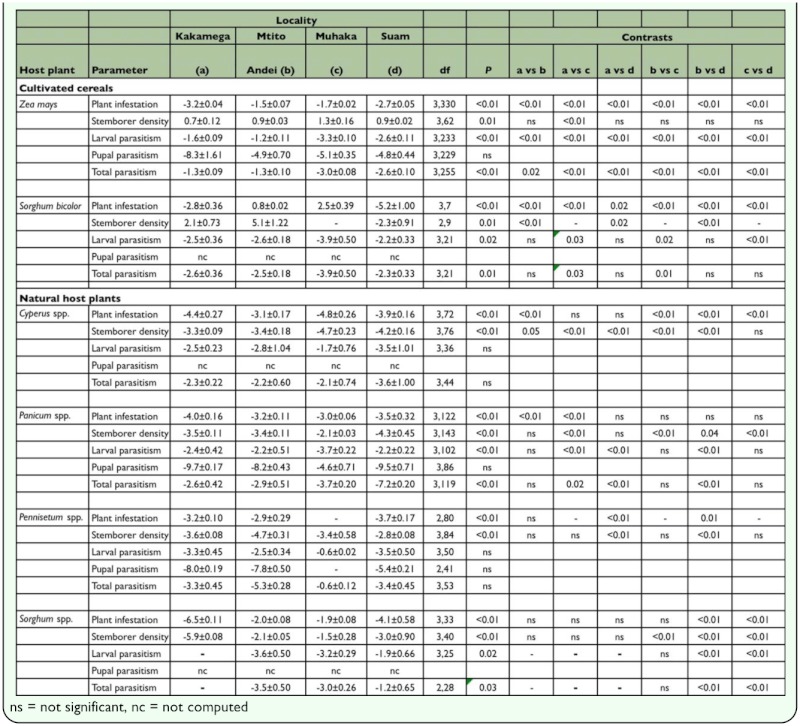
Least square means (±SE) of plant infestation, stem borer density and parasitism following binomial regression analysis across four localities (AEZs) in cultivated and natural habitats in Kenya

### Plant infestation, stem borer density and parasitism:

#### a) Based on locality

In maize, plant infestation, stem borer density, larval parasitism and total parasitism were significantly different amongst localities (Table 2). By contrast, pupal parasitism was not significantly different amongst localities. In sorghum, plant infestation, stem borer density, larval parasitism and total parasitism were significantly different amongst localities (Table 2). Pupal parasitism, however, was not computed due to insufficient data.

In *Cyperus* spp., whereas plant infestation and stem borer density were significantly different amongst localities (Table 2), larval and total parasitism were not. Pupal parasitism was not computed due to insufficient data. In *Panicum* spp., plant infestation, stem borer density, larval and total parasitism (Table 2) were significantly different amongst localities. By contrast, pupal parasitism was not significantly different amongst localities. In *Pennisetum* spp., although plant infestation and stem borer density were significantly different amongst localities (Table 2), larval and total parasitism were not. In *S. arundinaceum,* plant infestation, stem borer density, larval and total parasitism were significantly different amongst localities (Table 2). Pupal parasitism amongst localities, however, was not computed due to insufficient data.

**Table 3.**
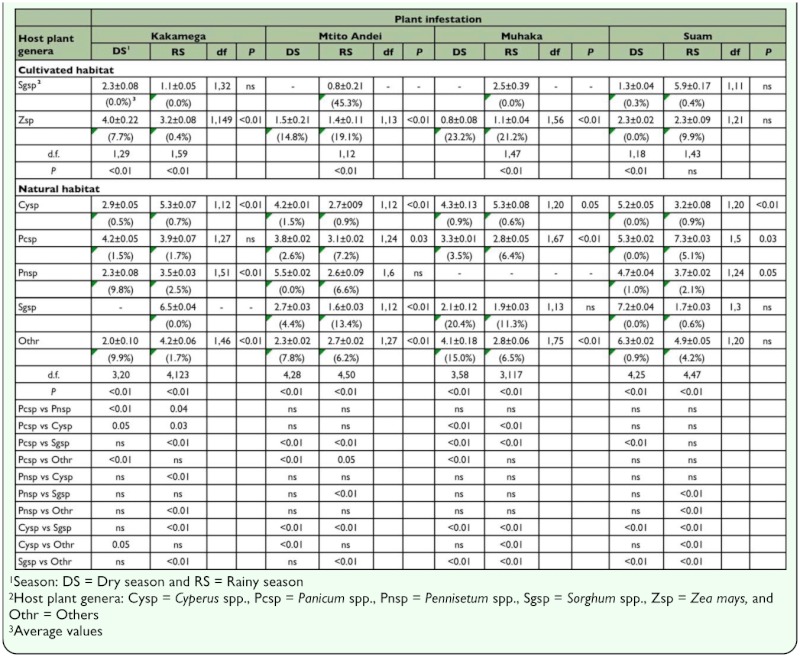
Least square means (±SE) following binomial regression analysis of plant infestation (%) during dry and rainy seasons in cultivated and natural habitats in four AEZs in Kenya

#### b) Based on season per locality

For cultivated habitats, with the exception of Suam, plant infestation was significantly different between maize and sorghum during the rainy season. Likewise, except for Suam, plant infestation was significantly different between seasons in maize (Table 3). Although stem borer density varied significantly between seasons in sorghum in Mtito Andei and Suam, it was not significantly different between seasons in maize in all localities (Table 4). Additionally, across localities, stem borer density was not significantly different between maize and sorghum during all seasons. For natural habitats, across localities, both plant infestation and stem borer density were significantly different amongst host plant genera during all seasons, and between seasons in at least two host plant genera (Tables 3 and 4). Across localities, seasons, and habitat types, plant infestation and stem borer density were generally highest in maize (Tables 3 and 4).

**Table 4.**
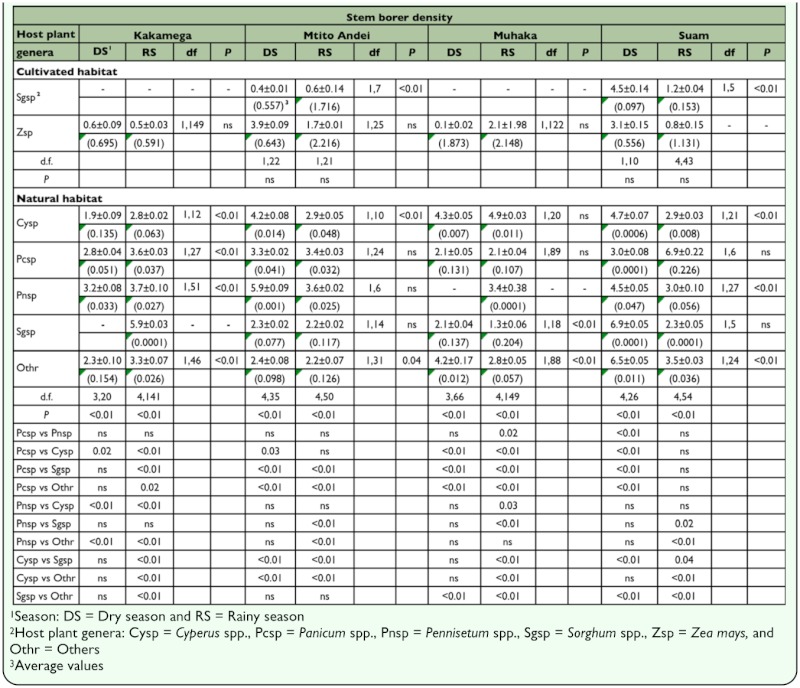
Least square means (±SE) following binomial regression analysis of stem borer density during dry and rainy seasons in cultivated and natural habitats in four AEZs in Kenya

In both habitats, with the exception of maize in Muhaka, larval parasitism was not significantly different between seasons on all host plants (Tables 5). Whereas in cultivated habitats larval parasitism was significantly different between maize and sorghum during the rainy season in only two localities (Mtito Andei and Muhaka), larval parasitism was significantly different mainly during the rainy season amongst various host plant genera in three different localities (Kakamega, Suam, and Muhaka). Across localities, seasons, and habitat types larval parasitism rates were generally highest in maize. In both cultivated and natural habitats, larval parasitism rates were highest during the rainy season. Across localities and habitat types, in addition to being generally low, pupal parasitism rates were neither significantly different among host plant genera nor across seasons in each host plant (Table 6). In cultivated habitats, with the exception of maize in Muhaka, total parasitism was not significantly different between seasons in maize and sorghum across localities (Table 7). In natural habitats, per host plant genera, total parasitism was not significantly different between seasons in all localities. Also, across habitats, total parasitism rate was highest in maize in cultivated habitats, particularly during the rainy season.

**Table 5.**
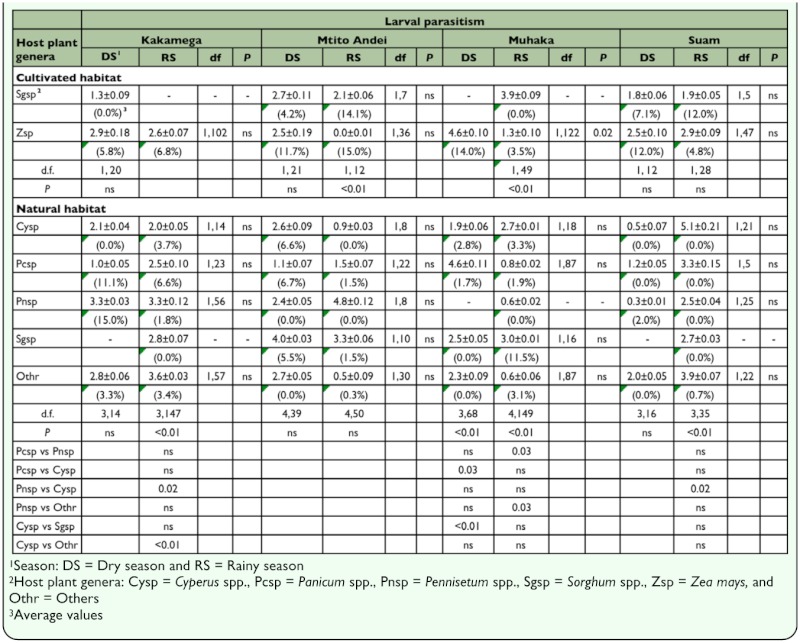
Least square means (±SE) following binomial regression analysis of larval parasitism (%) during dry and rainy seasons in cultivated and natural habitats in four AEZs in Kenya

## Discussion

Although highest total (larval and pupal) parasitism rate (<15.2%) was recorded in maize, stem borer parasitism was generally low in both cultivated cereals and wild host plants across localities and seasons. The results of larval and pupal parasitism in maize and cultivated sorghum align with parasitism rates previously reported by Skövgrad and Päts (1996) and Zhou et al. (2003). Likewise, larval parasitism recorded in wild host plants in this study fell within the range documented by Khan et al. (1997a) and Overholt et al. (1997). For the first time, this study provided stem borer pupal parasitism rates in wild host plants. However, because wild stem borers generally pupate outside plant stems (Mailafiya et al. 2009) it is very likely that current pupal parasitism rates were underestimated. Given that larval and pupal parasitism rates were highest across seasons in cultivated cereals, present results do not support the assumption by Bowden (1976), Conlong (1994), and Overholt (1998) that stem borer parasitism is higher in wild host plants than in cultivated cereals. Greater stem borer damage and amounts of larval frass usually produced in maize and sorghum compared to wild host plants (Ngi-Song et al. 1996; Ngi-Song and Overholt 1997), suggests higher herbivore host apparentcy in cultivated cereals than in wild host plants (Van Nouhuys and Via 1999; Barbosa and Calda 2007). Moreover, higher herbivore host densities in cultivated crops generally results in greater attraction/congregation and residence time of parasitoids (Sheehan and Shelton 1989; Connor and Cargain 1994; Umbanhowar et al. 2003) leading to higher host attacks.

**Table 6.**
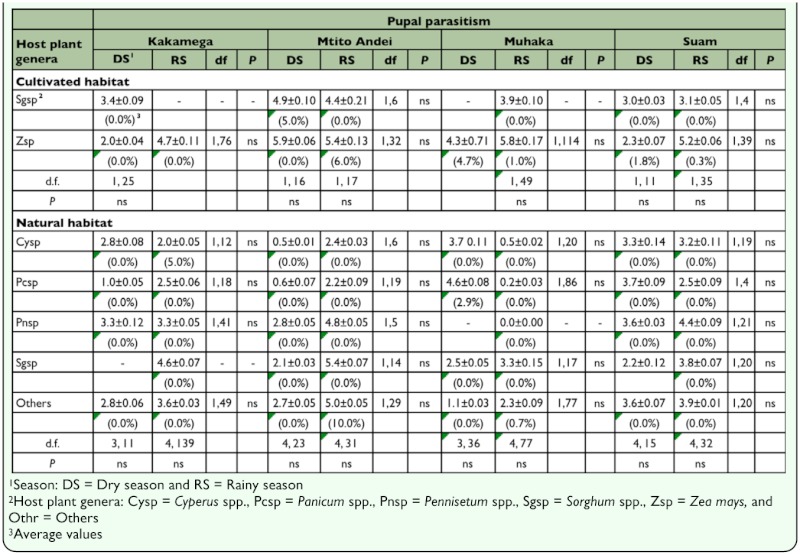
Least square means (±SE) following binomial regression analysis of pupal parasitism (%) during dry and rainy seasons in cultivated and natural habitats in four AEZs in Kenya

Low parasitoid searching efficiency in more complex habitats (i.e. natural habitats with greater host plant species composition and/or plant structures) (Andow and Prokrym 1990; Udayagiri and Welter 2000) might have contributed to low stem borer parasitism rates in wild host plants. For instance, Babendreier et al. (2003) found decreased searching efficiency to be responsible for lower parasitism of egg hosts by *Trichogramma brassicae* Bezdenko in non-crop plants than in maize. Low parasitism in wild host plants might have also been due to high mortality of parasitoids from toxic phytochemicals or their metabolites in the tissue and hemolymph of their herbivorous host (Ode 2006). Through sequestration, some herbivores utilize plant secondary chemicals in defense against their parasitoids to create enemy-free space (Stamp 2001; Nishida 2002). Singer and Stireman (2003) and Singer et al. (2004), for example, found that the woolly bear caterpillars, *Grammia geneura* (Lepidoptera: Arctiidae), feeding in two host plants that contain pyrrolizidine alkaloids, *Senecio longilobus* and *Ambrosia confertiflora,* was detrimental to the development of *Cotesia* sp. and two tachinid flies, *Exorista mella* and *Chetogena tachinomoides.* Meanwhile, information on both direct and indirect effects of plant toxicity in stem borer parasitoids is currently unavailable. Future investigations are needed, particularly because wild stem borers have been recovered from a wide range of host plant species (at least 66) (Le Ru et al. 2006a, b).

**Table 7.**
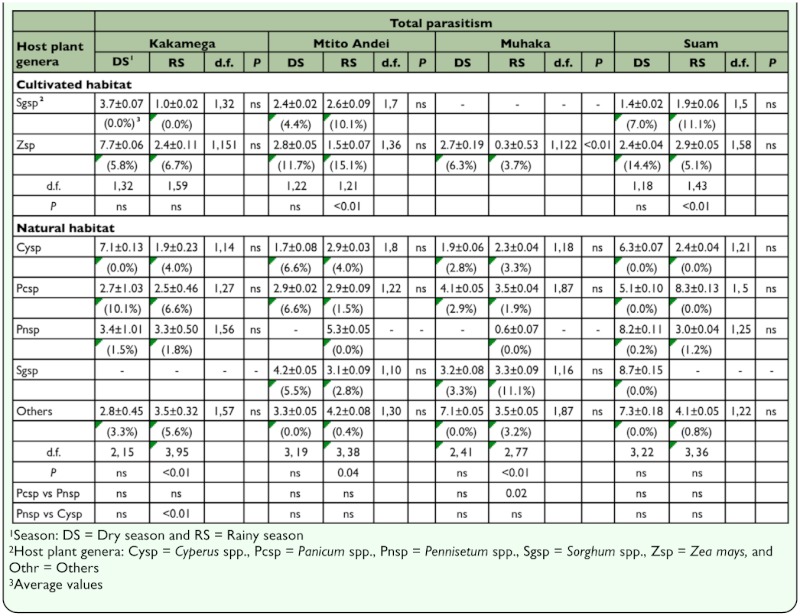
Least square means (±SE) following binomial regression analysis of total (larval and pupal) parasitism (%) during dry and rainy seasons in cultivated and natural habitats in four AEZs in Kenya

Low herbivore host densities across seasons in wild host plants in this study suggests low host encounter rates (Van Alphen 1993; Hemerik et al. 1993; Van Baalen and Hemerik 2008), and perhaps, frequent incidences of super or multiple parasitism (Van Alphen and Visser 1990; Godfray 1994) leading to the mortality of stem borers and associated parasitoids and/or low abundance and fitness of parasitoids. Studies by Agboka et al. (2002), confirmed that super parasitism of *Sesamia calamistis* Hampson eggs by *Telenomus* spp. within 24 hours after oviposition caused 40% mortality of the host. Additionally, Sétamou et al. (2005), showed the survivorship of parasitized stem borer larvae and parasitoid fitness to be much lower in wild host plants than in cultivated cereals. It will be interesting to have future studies elucidate the incidence and effects of super and multiple parasitism on stem borer parasitoid abundance and fitness in wild host plants.

Most host-parasitoid communities contain one or a few strong interactions, with the majority of parasitoids being opportunists that take advantage of available resources as they exert very weak parasitism rates (Polis and Strong 1996; Rodríguez and Hawkins 2000). In the case of stem borer parasitoid communities in Kenya, present results showed *C. flavipes* and *C. sesamiae* to be the key parasitoids species yielding strong interactions in regulating stem borer host populations in cultivated cereals (Mathez 1972; Skövgrad and Päts 1996; Ogol et al. 1998; Zhou et al. 2003). However, in wild host plants neither the two *Cotesia* species nor other parasitoid species exerted strong parasitism rates in stem borer hosts. This was attributed to the generally low stem borer host densities in wild host plants.

Consistent with the results in this study, Sétamou et al. (2005) also observed lower stem borer (an endogenous host) larval parasitism by *C. flavipes* in wild host plants than in cultivated crops. Benrey et al. (1998), likewise, using exogenous hosts recorded lower larval parasitism of *Pieris rapae* (L.) by *C. glomerata* (L.) in wild *Brassica* sp. and *Phaseolus* sp. than in their cultivated relatives. However, contrary to the above examples, Van Nouhuys and Via (1999) found higher larval parasitism of *P. rapae* by *C. glomerata* in wild than in cultivated cabbage. Additionally, on the sunflower moth, *Homoeosoma electellum* Hulst, with both exogenous (early instars feeding on pollen/floret) and endogenous (late instars as seed feeders) stages Chen and Welter (2007) demonstrated higher parasitism by *Dolichogenidea homoeosomae* Muesebeck in wild sunflowers than in their agricultural counterparts. Altogether, herbivore parasitism seemed to be high in wild host plants only in cases where: (1) parasitoid foraging efficiency was high owing to greater visual apparency of feeding hosts in less complex (structured) plants (Van Nouhuys and Via 1999), and (2) lack of structural refuge (i.e. hard seed coat) (Chen and Welter 2007) that offers little or no protection to herbivores against attacks.

Altogether, in spite of low stem borer parasitism in natural habitats in this study, natural habitats remain crucial for the sustenance of essential parasitoid diversity in cereal cropping ecosystems, as parasitoid diversity is higher in more diverse host plant communities (Mailafiya et al. 2010). Moreover, several abiotic and biotic factors affect the parasitization of stem borers. Irrespective of habitat type, stem borer parasitism is positively correlated with both parasitoid richness and abundance. Furthermore, being negatively correlated with temperature in natural habitats, parasitoids seem sensitive to harsh/extreme temperatures in this habitat, thereby either lowering their performance or decimating some of their populations (Mailafiya et al. 2010). Also, heavy rainfall, especially at higher altitudes, is detrimental to both stem borers (parastized or not) and parasitoids as well as their activities (Schulthess et al. 2001; Ndemah et al. 2003; Mailafiya et al. 2010)

In conclusion, evidence provided by field data in this study showed that stem borer parasitism was generally low during all seasons in wild host plants, and that cereal stem borers do not necessarily survive the off-season by remaining active in wild host plants. Low larval and pupal (or total) parasitism rates in cultivated cereals further reveal the need for enhancement of parasitoid effectiveness through biological control and/or habitat management practices to regulate cereals stem borer pests.
